# Modulation of endoglin expression in islets of langerhans by VEGF reveals a novel regulator of islet endothelial cell function

**DOI:** 10.1186/s13104-016-2142-z

**Published:** 2016-07-25

**Authors:** Claire E. Clarkin, Marwa Mahmoud, Bo Liu, Emmanuel O. Sobamowo, Aileen King, Helen Arthur, Peter M. Jones, Caroline P. Wheeler-Jones

**Affiliations:** 1 Diabetes Research Group, Division of Diabetes and Nutritional Sciences, School of Medicine, Kings College London, London, SE1 1UL UK; 2 Centre for Biological Sciences, University of Southampton, Building 85/Life Sciences, University Road, Southampton, SO17 1BJ UK; 3 Institute of Genetic Medicine, Newcastle University, London, NE1 3BZ UK; 4 Comparative Biomedical Sciences, The Royal Veterinary College, London, NW1 0TU UK

**Keywords:** Islets, Endoglin, VEGF, Hypoxia inducible factor-1 alpha

## Abstract

**Background:**

Endoglin/CD105 is an auxiliary receptor for transforming growth factor-β with established roles in vascular remodelling. It has recently been shown that heterozygous endoglin deficiency in mice decreases insulin secretion in an animal model of obesity, highlighting a potential role for endoglin in the regulation of islet function. We have previously identified two different populations of endoglin expressing cells in human and mouse islets which are: (i) endothelial cells (ECs) and (ii) islet mesenchymal stromal cells. The contribution of islet EC endoglin expression to islet development and sensitivity to VEGF is unknown and is the focus of this study.

**Results:**

In vitro culture of mouse islets with VEGF_164_ for 48 h increased endoglin mRNA levels above untreated controls but VEGF did not modulate VEGFR2, CD31 or CD34 mRNA expression or islet viability. Removal of EC-endoglin expression in vivo reduced islet EC area but had no apparent effect on islet size or architecture.

**Conclusion:**

EC-specific endoglin expression in islets is sensitive to VEGF and plays partial roles in driving islet vascular development, however such regulation appears to be distinct to mechanisms required to modulate islet viability and size.

## Background

Native pancreatic islets are vascularised by a dense network of microvessels that form an intra-islet portal system which provides insulin secreting β-cells with highly oxygenated blood and offers a rapid route for transport of insulin and islet hormones to reach their target tissues. The structural organisation of islets in which insulin-expressing β-cells are arranged in a specific pattern around blood vessels [1, 2] highlight the potential importance of endothelial to endocrine cross talk in the regulation of islet function. During embryonic development, reciprocal endothelial-endocrine signalling and the formation of functional blood vessels instruct pancreatic differentiation and morphogenesis [3–5].

Endoglin (Eng) is a homodimeric transmembrane glycol protein known to modulate cellular responses to ligands of the transforming growth factor (TGF)-β superfamily [6–9] and is expressed by vascular endothelial cells (ECs) [10–12]. Eng expression levels in the vasculature increase during blood vessel sprouting and decreased Eng expression leads to altered angiogenesis in vitro and aberrant vascular development and function in vivo [13–16]. Eng deficient (Eng −/−) mice die at E10.5–11.5 from cardiovascular defects due to inappropriate remodeling of mature vascular network [14, 16]. While Eng heterozygote (Eng±) mice are viable, they exhibit some of the vascular defects seen in humans with Eng haploinsufficiency [17]. A close relationship between Eng and pro-angiogenic vascular endothelial growth factor (VEGF) has been demonstrated in the differentiation of mouse embryonic stem cells (ESCs) in vitro, whereby hematopoietic commitment within VEGF receptor 2 (R2) positive precursor populations are characterised by Eng expression [18].

Previous studies suggest an involvement of Eng in the alteration of islet function associated with the development of diabetes. Thus, soluble plasma Eng levels have been reported to positively correlate with basal glycemia in patients with type 2 diabetes [19]. Mice with heterozygous Eng deficiency exhibit increased glucose following insulin injection versus wild type controls and decreases in insulin levels following high fat diet, were also evident, highlighting some importance for Eng in the regulation of islet function. [20]. Our previous work has described the presence of two distinct populations of Eng positive cells residing within human and mouse islets of langerhans which are (i) islet endothelial cells (ECs) and (ii) islet mesenchymal stromal cells (MSCs) [21]. We have reported a unique anti-angiogenic function of Eng expressing MSCs in islets but the role of EC-specific Eng expression in the regulation of islet vascular development remains unreported, and is the focus of this study. Specifically, we have investigated whether Eng expression in islets can be regulated directly by a proangiogenic factor such as VEGF in vitro. In addition, we have performed in vivo studies in which Eng was deleted specifically in ECs to assess the effects on islet phenotype during early postnatal pancreatic growth.

## Methods

Islets were isolated from male ICR mice (Harlan, Bicester, UK) as previously described by our group [21]. Islets from 4 to 6 mice were combined and used for each individual experiment which were repeated 3 times. For experiments using mitogens, media were refreshed every 24 h. Mouse recombinant VEGF_164_ was purchased from R and D systems (Minneapolis, MN).

Transgenic mice expressing inducible Cre ERT^2^ recombinase under regulatory control of the mouse VE-cadherin gene promoter region [22] were crossed with the floxed endoglin line [23] to generate Eng^fl/fl^; Cdh5(PAC) VE-Cadherin Cre-Ert2 mice (to be referred to as Eng-iKO^e^) *Cdh5*(PAC)Cre-ERT2 mice were obtained from Dr Ralf Adams (CRUK, London). *Eng* fl/fl *Cdh5*(PAC)Cre-ERT2 mice were backcrossed for 5 generations with C57BL/6 to generate an approximately syngeneic line. To activate Cre-ERT2, mice were injected subcutaneously with 0.5 mg tamoxifen at postnatal day (P)2 and P4 and pups sacrificed at P7. Genotyping was performed by PCR using genomic DNA template prepared from small tissue biopsies. Pancreata from 4 control and 4 mutant mice were collected for analyses.

Immuno-staining of fixed pancreatic sections for insulin, glucagon and CD34 has been described previously [11]. CD34-positive area was measured blind from light microscope images using Image J software. CD34 positive staining was quantified in 20 islets per mouse. For double labelling of Eng (Clone MJ7/18, provided as 0.5 mg/ml stock solution, eBioscience) and CD31 (Clone MEC13.3, provided as 15.625 μg/ml stock solution, BD Biosciences, Pharmingen) pancreas cryosections were fixed and incubated with primary antibodies [endoglin (1:100) and CD31 (1:25)] followed by donkey anti-goat alexa 594 and goat anti-rat alexa 48.

Mouse islets were isolated from 4 to 6 mice and combined for one experiment and each experiment was repeated 3 times. After isolation 150 islets counted, washed with PBS and lysed for RNA immediately. RNA was purified in preparation for RT-PCR according to manufacturer’s guidelines (Qiagen, Crawley, West Sussex, UK). Total cell RNA was reverse transcribed by incubating according to manufacturer’s instructions (Invitrogen, Paisley, UK) and standard PCR undertaken for amplification of mRNA from genes described in this manuscript (Primer sequences; [18]). Quantitative PCR each analysis using LC480 instrument (Roche, Burgess Hill, UK) and SYBR green PCR reaction mix (Roche, as above) contained a range of standards (known concentrations of same target sequence) and analyzed using LC480 analysis software. The standard curve was plotted which correlated cycle number with the amount of product formed after each cycle. mRNA levels were normalized to β-actin for each sample.

Viability of whole islets, was assessed by measuring ATP using the CellTiter-Glo luminescent cell viability assay (Promega, Southampton, UK) [22]. After isolation 20 islets where picked randomly and plated into a 96-well plate (6 wells/treatment). Each experiment was repeated 3 times.

## Results

### Effects of exogenous VEGF_164_ on endoglin expression and islet viability

**Fig. 1:**
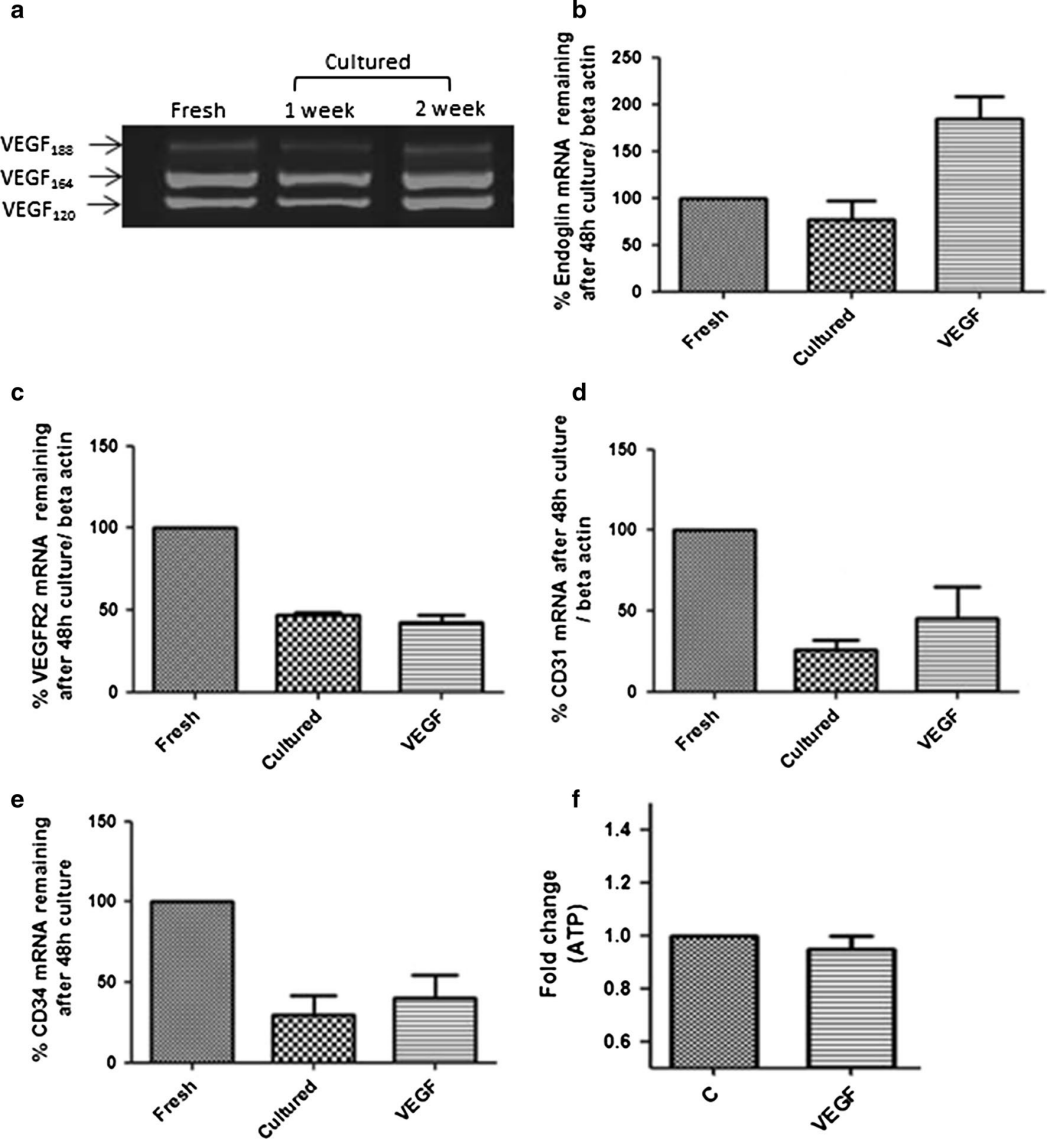
VEGF_164_ increases endoglin expression in cultured islets but does not modify islet viability. RNA was isolated from 30 mouse islets either directly following isolation (fresh) or following 1 or 2 weeks of culture. PCR was then undertaken for mRNA levels of VEGF splice variants (**a**). Real time PCR was also undertaken for Endoglin (**b**) VEGFR2 (**c**) CD31 (**d**) and CD34 (**e**) with RNA isolated from either freshly isolated islets or following 48 h culture in the presence and absence of VEGF_164_ (50 ng/ml). Data are normalised to β-actin and presented as % change with fresh islets normalised to 100 %. ATP levels in islets were measured following 48 h of VEGF_164_ (50 ng/ml) treatment (**f**). Data represent mean values + SEM and are from 3 separate mouse islet extractions

We have previously shown that Eng protein and mRNA expression in human and mouse islets is sustained during long-term culture of up to 2 weeks [21]. Consistent with this expression pattern we have now also found sustained expression of proangiogenic VEGF mRNA splice variants (120/164/188) in mouse islets cultured for up to 2 weeks (Fig. 1a). To establish whether exogenous VEGF_164_ could modulate Eng mRNA expression levels in islets, VEGF_164_ (50 ng/ml) was added to freshly isolated mouse islet cultures for 48 h (media refreshed after 24 h) and RNA isolated. Quantitative PCR showed an increase in Eng mRNA following exposure to VEGF_164_ above cultured islet controls (Fig. 1b). This observation is in contrast to the mRNA levels of other endothelial cell markers including VEGFR2, CD31 and CD34 which are all reduced after 3 days of culture and are unmodified by VEGF treatment (Fig. 1c, d, e). To investigate how such changes in Eng mRNA may impact islet viability, freshly isolated mouse islets were treated with 50 ng/ml of VEGF_164_ for 48 h and islet ATP levels were measured as an indicator of mitochondrial function and cell viability. VEGF had no apparent effect on islet ATP content (Fig. 1f) demonstrating that the modulation of EC specific endoglin mRNA expression by VEGF is not linked to changes islet viability in vitro.

### Islet architecture in endothelial cell specific Eng knock out mice

**Fig. 2:**
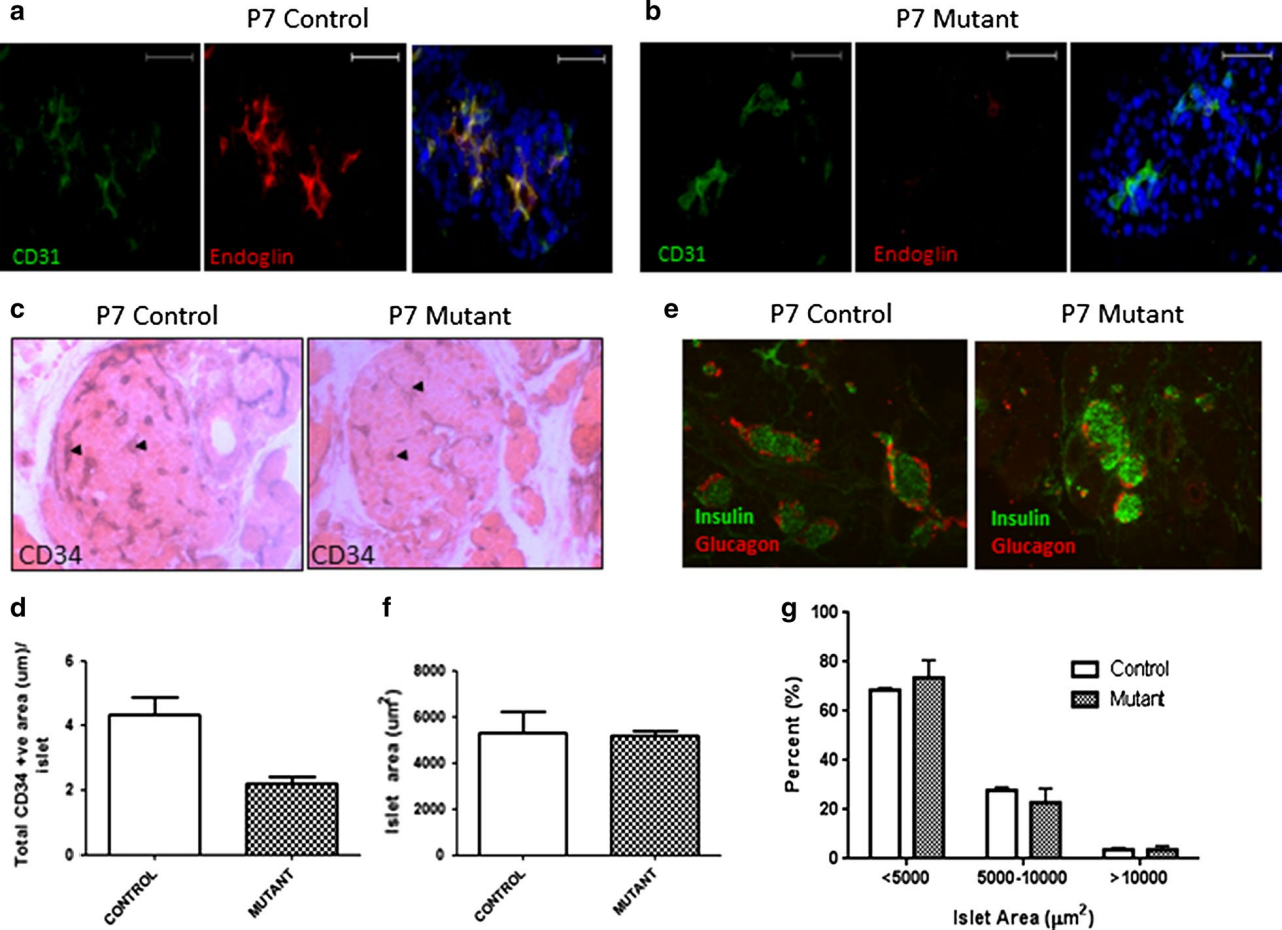
Loss of endothelial cell-specific endoglin expression reduces total endothelial cell area per islet. Pancreases of endoglin fl/fl VE-Cadherin Cdh5(PAC)Cre-ERT^2^ (mutant) mice and endoglin fl/fl (control) P7 neonates were collected, processed and co-labelled for CD31 and Endoglin to verify ablation of endoglin by ECs of mutant mice (**a**, **b**). Immunohistochemical analysis of CD34-positive staining was undertaken using HRP-DAB (eosin counterstain; **c** *black arrows*. Sections were also double labelled for insulin (fluorescein) and glucagon (*texas red*; **d**). Measurements of islet vascular density taken from ~20 islets/per mouse and expressed as CD34-positive area (µm; **e**) per islet, total islet area (µm^2^; **f**), Islet size distribution was also analysed (%; **g**). Data are mean values (n = 4 animals per group) + SEM

To investigate the potential involvement of EC-specific Eng in modulating the islet phenotype in vivo we used an inducible EC-specific endoglin knock out mouse; Eng-iKO^e^. Efficient Eng deletion in ECs following tamoxifen treatment of Eng-iKO^e^ animals and litter mate wild type controls was first verified by double fluorescent labelling of Eng and CD31. CD31-positive cells within the islets of Eng-iKO^e^ were devoid of Eng staining when compared to control wild type animals (Fig. 2a, b). In pancreases from Eng-iKO^e,^ measurement of EC area per islet (as assessed by positive CD34 staining/islet area) was lower than that in islets from control mice (Fig. 2c, e), although CD34 staining was still detectable in mutant animals. In contrast, islet size and insulin and glucagon staining (for β/α-cell identification respectively) were not different in control versus mutant pancreases (Fig. 2d, f). Classification of islets by size (i.e. <5000, 5000–10,000 and >10,000 um^2^), also revealed no differences in the distribution of islet sizes between wild type and Eng-iKO^e^ mice (Fig. 2g), confirming the lack of effect of EC-specific Eng deletion on islet morphology.

## Discussion

Pancreatic islets are highly vascularised, which is important not only during development but also in their ability to quickly secrete insulin in response to changes in blood glucose. In adults, VEGF is highly expressed and secreted by insulin-producing β-cells and is thought to contribute to the rich islet vascularisation [1–3]. Today, considerable effort is currently being devoted to establish the effects of pro-angiogenic factors such as VEGF on the success of islet graft vascularization and survival of newly transplanted islets. In our study we found that addition of VEGF_164_ to freshly isolated islets for 48 h did not however influence islet viability, or the expression of endothelial cell markers such as VEGFR2, CD31 and CD34.

The role of VEGF in improving islet transplantation outcomes is currently disputed; overexpression of VEGF in transplanted mouse islets caused increased vascularisation and increased blood flow to newly transplanted islets, resulting in improved recipient glucose tolerance, and increased β-cell survival [1]. However in another study VEGFs removal from β-cells did not modify β-cell mass, but lead to insufficient islet vascularisation, resulting in defective insulin secretion [4].

VEGF is highly responsive to decreasing oxygen levels and a hypoxia-responsive element downstream of the main transcription start site of the Eng gene has also been functionally characterised with hypoxic conditions shown to up-regulate the Eng gene promoter, transcript and protein levels [24]. The high levels of VEGF, and Eng seen in our freshly isolated mouse islets, are predicted to be a consequence of the islet isolation procedure and following the loss contact with the systemic pancreatic blood supply. Given the high levels of these genes expressed by freshly isolated islets it was perhaps unsurprising that addition of exogenous VEGF served to further increase Eng mRNA levels by potentially exacerbating the hypoxic response. Whether direct modulation of Eng expression may provide a novel means to improve islet transplant outcomes via a positive influence on the vasculature is currently unclear, but regulation of its expression in islets may provide a novel route for possible interrogation.

To further investigate how modifications in EC specific-Eng expression may have implications for islet development in vivo, Eng was deleted from postnatal day 2 until postnatal day 7 in all VE-Cadherin positive cells. We found that loss of EC specific Eng reduced EC number but did not impact islet growth and architecture at postnatal day 7. A recent study has shown that Eng is not required for the initial process of vasculogenesis, but is required for VEGF-induced angiogenesis, which could explain why deletion of Eng did not completely ablate all islet blood vessels [25]. To date, the role of EC-Eng expression during the tightly regulated steps of pancreatic development, and its control by VEGF, remains unclear but we have highlighted for the first time a direct involvement of Eng in islet vascular growth in vivo. In addition, given our in vitro observations whereby addition of VEGF can upregulate Eng mRNA, it is expected that expression of Eng in vivo will be tightly coordinated by the islets allowing for rapid and sufficient angiogenesis at times of growth and remodelling.

Our in vivo studies demonstrated altered EC number following deletion of Eng expression which were independent of changes in islet size and architecture at postnatal day 7. An uncoupling of EC and β cell behaviour has been described with recent publications showing that changes in β-cell mass and insulin resistance were associated with modifications in vascular dilation of pre-existing vessels but not through increased angiogenesis [26]. Furthermore, when VEGF was deleted postnatally in islets using β-cell specific DOX-inducible transgenics and a VEGF trap (Flt-1) EC regression and loss of EC fenestrae at 8–12 weeks of age was described. However, although islet hypovascularisation caused intraislet hypoxia in these animals, blood glucose was only marginally elevated and did not affect insulin gene expression, pancreas insulin content or beta cell proliferation rate [27]. Additionally, in a study where VEGF was deleted in adult beta- cells although there was a 50 % reduction in the adult islet vasculature measured, islets were capable of maintaining their morphology, gene expression and beta cell mass, highlighting the remarkable ability islets have to survive and function when intraislet endothelial cells are reduced [28]. Similarly, our observations that the reduction in EC number following Eng deletion did not influence the size or architecture of individual islets, nor the overall beta cell mass as assessed by islet area, is further evidence for an uncoupling of endothelial/beta cell behaviour. As the vasculature is still detectable in islets when Eng is removed in combination with the published role for Eng in regulating angiogenesis not vasculogenesis [25] it is possible that there remains sufficient vascularisation necessary for normal islet growth within this particular stage of development. Since previous studies suggest that the functional beta cell mass is the primary predictor of metabolic control [27–29] the Eng deletion is alone unlikely to exert a metabolic phenotype, although this should be confirmed in future studies.

## Conclusions

We have shown that in isolated islets VEGF can directly increase Eng mRNA expression levels and that Eng is required in part for islet EC growth during development. Therefore, modulation of Eng expression may provide a novel route to improve islet graft revascularisation. Our findings showing no effect of EC specific-Eng deletion on islet phenotype may point towards independent mechanisms driving islet vascularisation and islet growth in early development. Future investigations interrogating the relationship between islet ECs and beta-cells are required to better understand the pathways involved in promoting and maintaining islet vascular function which could be manipulated in clinical settings.
